# Potential coordination role between *O*-GlcNAcylation and epigenetics

**DOI:** 10.1007/s13238-017-0416-4

**Published:** 2017-05-09

**Authors:** Donglu Wu, Yong Cai, Jingji Jin

**Affiliations:** 1 School of Life Sciences, Jilin University, Changchun, 130012 China; 2 National Engineering Laboratory for AIDS Vaccine, Jilin University, Changchun, 130012 China; 3 Key Laboratory for Molecular Enzymology and Engineering, The Ministry of Education, Jilin University, Changchun, 130012 China

**Keywords:** *O*-GlcNAcylation, post-translational modification, histone modification, epigenetics

## Abstract

Dynamic changes of the post-translational *O*-GlcNAc modification (*O*-GlcNAcylation) are controlled by *O*-linked β-N-acetylglucosamine (*O*-GlcNAc) transferase (OGT) and the glycoside hydrolase *O*-GlcNAcase (OGA) in cells. *O*-GlcNAcylation often occurs on serine (Ser) and threonine (Thr) residues of the specific substrate proteins via the addition of *O*-GlcNAc group by OGT. It has been known that *O*-GlcNAcylation is not only involved in many fundamental cellular processes, but also plays an important role in cancer development through various mechanisms. Recently, accumulating data reveal that *O*-GlcNAcylation at histones or non-histone proteins can lead to the start of the subsequent biological processes, suggesting that *O*-GlcNAcylation as ‘protein code’ or ‘histone code’ may provide recognition platforms or executive instructions for subsequent recruitment of proteins to carry out the specific functions. In this review, we summarize the interaction of *O*-GlcNAcylation and epigenetic changes, introduce recent research findings that link crosstalk between *O*-GlcNAcylation and epigenetic changes, and speculate on the potential coordination role of *O*-GlcNAcylation with epigenetic changes in intracellular biological processes.

## Introduction

A large number of intracellular proteins often undergo post-translational modifications. As one of the highly dynamic, inducible, and reversible post-translational modification, *O*-GlcNAc on Ser/Thr residues of nuclear, cytoplasmic, and mitochondrial proteins is ubiquitous in eukaryotic cells (Wells and Hart, 2003; Bullen et al., 2014). Dynamic changes of *O*-GlcNAcylation are controlled by OGT and OGA (Gao et al., 2001). The sugar nucleotide UDP-GlcNAc serves as a donor for *O*-GlcNAc addition to nucleocytoplasmic proteins whereas OGA removes the *O*-GlcNAc group from proteins (Mailleux et al., 2016). Numerous studies have demonstrated that *O*-GlcNAcylation is involved in many fundamental cellular processes, including gene transcription (Ha and Lim, 2015), cell signaling (Cassey, 1995), and apoptosis (Butkinaree et al., 2008). More than those, many transcriptional factors, tumor suppressors, oncogenes, and cell receptors have been found to be tightly associated with *O*-GlcNAcylation in tumorigenesis (Bond and Hanover, 2015; Charoensuksai et al., 2015). Recently, accumulating data reveal that *O*-GlcNAcylation is frequently related to epigenetic changes. For example, *O*-GlcNAcylation at serine 112 site of histone H2B (H2BS112) by OGT stimulates H2B at lysine 120 (H2BK120) ubiquitination that further activates gene transcription such as ring finger protein 20 (RNF20) (Fujiki et al., 2011; Nakamura et al., 2011), suggesting the existence of complicated coordinative role between *O*-GlcNAcylation and the other histone post-translational modifications. In this review, we focus on the *O*-GlcNAcylation by OGT, summarize the current understanding of the crosstalk between *O*-GlcNAcylation and epigenetic changes in cells.

## Intracellular *O*-GlcNac modification is regulated by OGT and OGA

As is well known, *O*-GlcNAcylation is the process of adding *O*-GlcNAc via an *O*-linkage to Ser or Thr residues of intracellular proteins. Thousands of *O*-GlcNAcylated proteins have been found to implicate in the regulation of core processes including cellular metabolism, growth, and other important function (Copeland et al., 2008; Hanover et al., 2012). In cells, *O*-GlcNAcylation is often referred to as a nutrient sensor. The sensitivity of nutrient is based on the levels of UDP-GlcNAc, the donor substrate for protein *O*-GlcNAcylation, while UDP-GlcNAc is the end product of the hexosamine biosynthetic pathway (HBP). Thus, the levels of UDP-GlcNAc in cells are closely associated with flux through the HBP (Marshall et al., 1991). According to the current reports, OGT and OGA are the only two enzymes to control intracellular *O*-GlcNAcylation.

**Figure 1:**
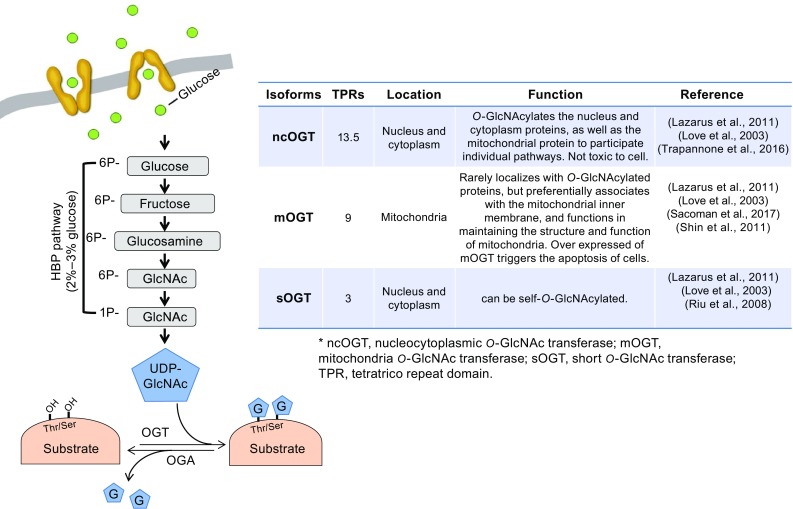
**The process of** ***O*** **-GlcNAc modification and the molecular structure of OGT isoforms**. A range of 2%–3% glucose uptake by the cells goes through the HBP pathway, experiences a series of modification and synthesize UDP-GlcNAc, the substrates of *O*-GlcNAc reaction. OGT/OGA are the enzymes in charge of add/remove UDP-GlcNAc at Ser or Thr residues respectively to control the balance of intracellular *O*-GlcNAcylation level. The molecular structure of 3 isoforms of OGT is highly conserved. By alternative splicing, 3 isoforms of OGT including nucleocytoplasmic isoform (ncOGT, 116 kDa), the mitochondrial isoform (mOGT, 103 kDa), and the short isoform (sOGT, 75 kDa) are produced.

OGT is highly conserved from *Caenorhabditis elegans* to human. Functional domain studies have been clarified that full length of OGT is composed of two important regions: an N-terminal tetratricopeptide-repeat (TPR) super-helical structure and a C-terminal catalytic domain (Jinek et al., 2004; Kreppel and Hart, 1999). Wherein, TPR domain is involved in substrate recognition through serving as protein:protein docking sites for substrate targeting proteins (Lubas and Hanover, 2000), and C-terminal catalytic domain binds to donor UDP-GlcNAc and is responsible for catalyzing the substrate targeting proteins by adding *O*-GlcNAc group (Lazarus et al., 2011). In cells, three different transcripts encoding nucleocytoplasmic isoform (ncOGT, 116 kDa), the mitochondrial isoform (mOGT, 103 kDa), and the short isoform (sOGT, 75 kDa) are produced by alternative gene splicing. C-terminal of these isoforms is completely the same, and only differs in the number of N-terminal TPRs (Hanover et al., 2003). As shown in Fig. 1, 13.5, 9, and 3 TPRs are contained in ncOGT, mOGT, and sOGT, respectively. The different localizations and the number of TPRs in each isoform suggest that each OGT isoform possesses its own substrate specificity and function (Lazarus et al., 2011; Love et al., 2003; Trapannone et al., 2016; Sacoman et al., 2017; Shin et al., 2011; Riu et al., 2008).

Another important enzyme that affects intracellular *O*-GlcNAcylation is *O*-GlcNAc hydrolase OGA. OGA was initially isolated from crude cellular extract, and it catalyzes hydrolytic cleavage of *O*-GlcNAc from proteins (Gao et al.,  2001). Full-length OGA is composed of N-terminal N-acetyl-β-D-glucosamindase domain and C-terminal with acetyltransferase-like (*AT*) domain (Toleman et al., 2004). Alternative gene splicing results in two different lengths of OGA isoforms: one isoform has a 916 amino acid (OGA-L) and predominantly localizes in the cytoplasm, while the other only has a 677 amino acid (OGA-S), and localizes in nuclear and lipid-droplet (Comtesse et al., 2001).

In view of the OGT and OGA are the only enzymes that involved in *O*-GlcNAc post-translational modification in cells, it is not difficult to understand that both enzymes are essential to cell growth, ontogeny, and survival in mammals. As reported, OGT modulates the somatic cell function and embryo viability through *O*-GlcNAc modification of X-chromosome-linked proteins in mice (O’Donnell et al., 2004). Furthermore, global *O*-GlcNAcylation of rat brain protein is relatively high at early development stage, and gradually decreases during the development, suggesting the coordinative role between OGT and OGA in cell growth and differentiation (Liu et al., 2012).

## Mode of OGT in cells

Although the expression level of OGT in the pancreas and brain is higher than other tissues (Kreppel et al., 1997), OGT is ubiquitously expressed in all tissues, showing the essential role of OGT in cells. In line with this view, numerous studies have confirmed the broad presence of *O*-GlcNAc modification involved in many fundamental cellular processes such as cell morphogenesis, cell signaling, apoptosis, and transcription (Lazarus et al., 2006). It is worth noting that, in addition to free form, OGT can also be assembled in complex to participate in intracellular biological processes.

Ten-eleven translocation (TET) family enzymes including TET1, TET2, TET3 catalyze the conversion of 5-methylcytosine (mC) to 5-hydroxymethylation (hmC) (Ito et al., 2011). Recent studies have found that each enzyme of TET family (TETs) can be complexed with OGT to play a cellular function. The complexes of TET2/TET3 with OGT can *O*-GlcNAcylate the host cell factor 1 (HCF1), a shared subunit of the methyltransferase SET1/COMPASS and histone acetyltransferase NSL complexes, therefore impact the histone H3K4 tri-methylation (H3K4me3) via SET1/COMPASS (Deplus et al., 2013). In-depth study demonstrated that the TET3-OGT complex enhances the recruitment of OGT to chromatin through its stabilization (Ito et al., 2014). TET1, being no exception, can interact with OGT, and the TET1-OGT complex regulates the recruitment of OGT to chromatin and TET1 activity in embryonic stem cells (Vella et al., 2013). Thus, we speculate that the TETs-OGT crosstalk not only help OGT to raise in chromatin, but also regulates gene transcription by affecting histone modification and chromatin structure. In addition to TETs proteins, the function of OGT is also connected to other intracellular proteins. For example, a heterotrimeric complex formed by URI, OGT, and PP1γ regulates cellular *O*-GlcNAcylation in response to metabolic stress (Burén et al., 2016). However, in C2C12 skeletal muscle cells, OGT and AMPK (AMP-activated protein kinase) cooperatively regulate nutrient-sensitive intracellular metabolism, growth, and proliferation (Bullen et al., 2014). Furthermore, a stable complex of MLL5 (mixed lineage leukemia 5), OGT, and USP7 (ubiquitin specific protease 7) was verified by Zhang Y’s group (Ding et al., 2015), and the coordinative expression between three proteins was observed in primary cervical adenocarcinomas. The above results suggest that OGT participates in intracellular different biological processes via forming different protein complexes.

**Figure 2:**
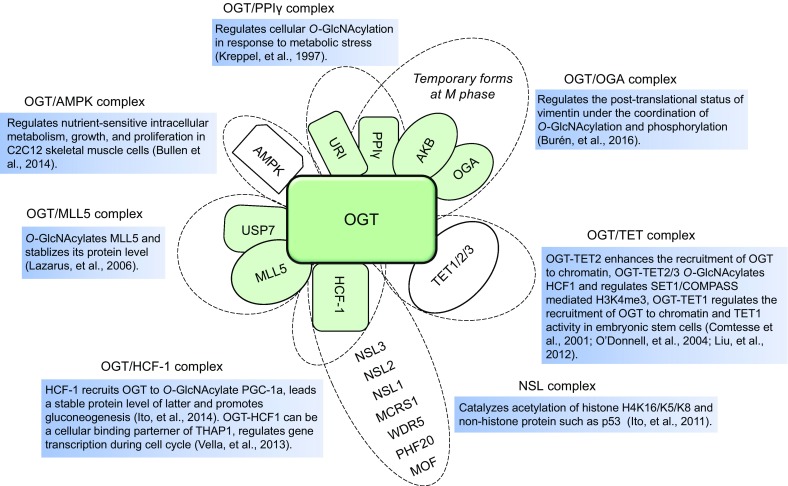
**Regulation and function of OGT-mediated protein complexes**

We previously described a second human MOF (males absent on the first)-containing histone acetyltransferases NSL (non-specific lethal) complex, which comprises 9 subunits and can acetylate histone H4 at lysine 16 (K16), 5 (K5), and 8 (K8) (Cai et al., 2010). Although the function of OGT in NSL complex is unclear, the coordinative role of OGT and HCF1 has been reported. For instance, *O*-GlcNAcylation by OGT to the specific repeats region of HCF-1 may provide instructions for the HCF-1 proteolysis (Capotosti et al., 2011). In addition, the interaction of OGT/*O*-GlcNAcylation and HCF1 stabilizes PGC-1α, a master regulator of gluconeogenesis. Knockdown both OGT and HCF1 significantly decreases the expression of gluconeogenic genes induced by PGC-1α in fed mice, suggesting the OGT/HCF1 sub-complex-mediated cooperative regulation in gluconeogenesis (Ruan et al., 2012). Also, OGT and HCF1 were characterized as major cellular binding partner of THAP1, a sequence-specific DNA binding factor (Mazars et al., 2010). Besides those stable complexes, OGT can also form a temporary complex with OGA, PP1γ, and Aurora B to regulate the post-translational status of vimentin under the coordination of *O*-GlcNAcylation and phosphorylation (Slawson et al., 2008), indicating the manifold functions of OGT assembled in complexes (Fig. 2).

## Crosstalk between *O*-GlcNAcylation and epigenetic mechanisms

**Figure 3:**
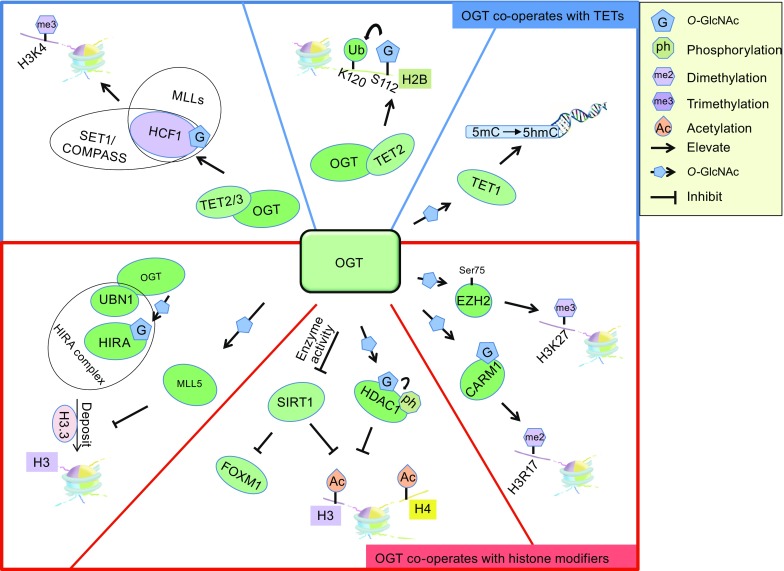
**Crosstalk between** ***O*** **-GlcNAcylation and chromatin remodelers**. Extensive connections of *O*-GlcNAcylation with chromatin modifiers are detected in cells

Changes in chromatin structure are mainly caused by N-terminal tails of histone modification and ATP-dependent chromatin remodelers (Gerhold and Gasser, 2014; Längst and Manelyte, 2015; Jin et al., 2005). These complexes regulate the gene expression by epigenetic mechanism in most cellular biological processes (Bannister and Kouzarides, 2011). Recent research evidence shows that *O*-GlcNAcylation by OGT is often implicated in chromatin-mediated processes in a coordinated manner (Fig. 3). According to OGT is an important and indispensable enzyme in cell metabolic pathway, unbalanced *O*-GlcNAc modification on substrate proteins leads to various kinds of diseases, such as diabetes, neurologic disorders, cardiovascular disease, and cancer (Gao et al.,  2001). Especially, high *O*-GlcNAc modification has been found in breast cancer (Caldwell et al., 2010; Gu et al., 2010; Krzeslak et al., 2012), prostate cancer (Lynch et al., 2012; Kamigaito et al., 2014; Gu et al., 2014), lung and colon cancer (Mi et al., 2011), esophageal cancer (Qiao et al., 2012), and hepatocellular carcinoma (Zhu et al., 2012). More importantly, numerous tumor-related proteins are also modified and regulated by OGT, such as oncoprotein c-Myc (Chou et al., 1995; Itkonen et al., 2013) and tumor suppressor gene p53 (Yang et al., 2006; de Queiroz et al., 2016). The role of OGT/*O*-GlcNAcylation in some important signal transduction pathways cannot be ignored. In cholangiocarcinoma (CCA) cells, matrix metalloproteinases (MMP)-mediated migration and invasion of CCA cells are regulated by *O*-GlcNAcylation through affecting the nuclear translocation of NFκB (Phoomak et al., 2016).

### Coordination of *O*-GlcNAcylation and TET proteins

Given that TETs implicated in controlling genome-wide DNA methylation and cellular differentiation, the impact of TETs proteins in tumorigenesis has been investigated (Shi et al., 2013; Fu et al., 2013). Subsequent research found that TET1 is a fusion partner of mixed-lineage leukemia (MLL)-rearranged acute myeloid leukemia (AML) (Ono et al., 2002; Lorsbach et al., 2003). More importantly, TET genes, especially TET2 are frequently mutated in various cancers, including myelodysplastic syndrome (MDS), cohort of chronic myelomonocytic leukemia (CMML), primary and secondary AML, blastic plasmacytoid dendritic neoplasm (Scourzic et al., 2015; Delhommeau et al., 2009; Langemeijer et al., 2009; Tefferi et al., 2009). However, transcriptional regulation of TETs might be associated with its interplay with histone modifications. Supporting this speculation, OGT-dependent H2BS112 *O*-GlcNAcylation is regulated by TET2 in embryonic stem cells (Chen et al., 2013). While genome-wide analysis confirms that *O*-GlcNAcylation of H2BS112 is widely distributed over chromosomes, and is frequently co-localizes with H2BK120 mono-ubiquitination (Fujiki et al., 2011), suggesting the complexity among TET2, *O*-GlcNAcylation, and H2BK120 ubiquitination.

As we mentioned earlier, TETs proteins function to recruit OGT to chromatin. Thus, it is not difficult to understand that knocking down TETs proteins declines the *O*-GlcNAcylation of chromatin proteins. In mouse embryonic stem (ES) cells, TET1-OGT interaction links to multiple chromatin regulators including Sin3A and NuRD complexes (Shi et al., 2013). Follow-up studies clarify that TET1 with those chromatin regulators together commands the level of local 5hmC to regulate the expression of target genes (Wu et al., 2011; Williams et al., 2011; Yildirim et al., 2011), suggesting the existence of crosstalk between TET1-OGT and TET1-chromatin remodelers in gene transcription. Consistent with this conjecture, knocking down either TET1 or OGT in mouse ES cells leads to reduced genomic targeting of both Ezh2 (enhancer of Zeste 2 polycomb repressive complex 2 subunit, a subunit of PRC2 complex) and Sin3A, indicating the importance of TET1-OGT coordination in repressing developmental genes (Shi et al., 2013). In *drosophila*, polycomb group gene *super sex comb* (*sxc*) that regulates the bithorax complex is turned out to encode OGT, and play essential role in polycomb repression (Gambetta et al., 2009; Ingham, 1984). Confirming to this conclusion, TET1-OGT complex represses gene transcription through interaction with Sin3A/HDAC1/Sirt1 and PCR2/Ezh2 complexes (Hardivillé and Hart, 2012; Williams et al., 2012). Similar effects are reflected in TET2-OGT or TET3-OGT interaction. They can target OGT to transcriptional start site (TSS), and in collaboration with HCF1 recruit SET1/COPASS complexes, therefore activate gene transcription through histone H3K4me3 at target promoters (Deplus et al., 2013). TETs-OGT/*O*-GlcNAcylation is involved in some cancer development. In 5-fluorouracilresistant colon cancer cells (SNUC5/5-FUR), highly expressed OGT binds to TET1 and recruits to the Nrf2 (nuclear factor erythroid 2-related factor 2) promoter region, suggesting the role of OGT in TET1-mediated Nrf2 expression (Kang et al., 2016). Taken together, TETs proteins in collaboration with OGT play a critical role in regulating chromatin structure and gene transcription.

### Coordination of *O*-GlcNAcylation and other modifiers on histones

Intracellular biological processes are extremely complex, and are often regulated by two or more histone modifiers in a co-ordinated manner. *O*-GlcNAcylation-mediated mechanism is no exception, often in collaboration with other histone modifications to regulate the biological processes in cells. Besides all four histones (H2A, H2B, H3, and H4) can be *O*-GlcNAcylated by OGT (Zhang et al., 2011; Sakabe et al., 2010) (Table 1), the accurate modified sites are also identified gradually. Genome-wide studies confirm that *O*-GlcNAcyaltion of histone H2A at Ser40 is dramatically changed during the differentiation in mouse trophoblast stem cells (Hirosawa et al., 2016). However, *O*-GlcNAcylation on histone H2A at Thr101 can relax chromatin structure through destabilizing H2A-H2B dimer (Lercher et al., 2015). On the other hand, occurrence of *O*-GlcNAcylation on histone variant H2AX at Ser139 is often detected in DNA damage foci (Chen and Yu, 2016). Obviously, *O*-GlcNAcylation on histone H2A at different sites is tightly associated with different intracellular functions. In cells, *O*-GlcNAcylation on histone H2BS112 may preserve a stable chromatin at the early stage of adipocyte differentiation, thus repressing gene transcription in cell fate (Ronningen et al., 2015). And H2BS112-*O*-GlcNAcylation facilitates H2B at K120 ubiquitination, the latter further acts as a platform recruiting the SET1/COMPASS complex binding to histone H3, thereby activates gene transcription through histone H3K4me3 (Deplus et al., 2013).

**Table 1:** *O*-GlcNAcylation sites and functions of histone tails. *O*-GlcNAc modification is observed in all four histones (H2A, H2B, H3 and H4) as well as histone variants H3.3 at indicated sites.

Histones	*O*-GlcNAcylated sites	Functions	References
**H2A**	Ser40	Tightly relates with the differentiation in mouse trophoblast stem cells	(Hirosawa et al., 2016)
	Thr101	Destabilizes H2A-H2B dimer, further relaxes the structure of chromatin	(Lercher et al., 2015)
**H2AX**	Ser139	Co-localizes with DNA damage foci, may function in DNA damage repair	(Chen and Yu 2016)
**H2B**	Ser112	Preserves a stable chromatin and represses gene transcription at the early stage of adipocyte differentiation Promotes H2BK120 ubiquitination, participates the regulation of H3K4me3 and gene transcription	(Ronningen et al., 2015) (Deplus et al., 2013)
	Ser36	May be a part of the histone code	(Sakabe et al., 2010)
**H3**	Thr32	Increases the phosphorylation of Thr32, Ser28, and Ser10, which are the specific mark of mitosis	(Zhang et al., 2011) (Fong et al., 2005)
	Ser10	Competitively reduces the levels of H3S10 phosphorylation, therefore regulates the pathway that H3S10P involved in, such as passing the G_2_-M phase check point, regulating the H4K16ac	(Zhang et al., 2011)
**H4**	Ser47	May be a part of the histone code	(Sakabe et al., 2010)

Ser40/139/112/10/36/47, serine residues 40/139/112/10/36/47; Thr101/32, threonine residue 101/32; H2BK120, H2B lysine 120; H3K4me3, H3 lysine 4 tri-methylation; H4K16ac, H4 lysine 16 acetylation

Interestingly, competitive modification between *O*-GlcNAcylation and phosphorylation on Ser/Thr residues of substrate proteins may be closely related with functional switch. For example, higher *O*-GlcNAcylated H3 at Thr32 is observed during interface than mitosis. Further research demonstrates that *O*-GlcNAcylated Thr32 reduces mitosis-specific phosphorylation of Thr32, Ser28, and Ser10 on H3, suggesting the switching function of *O*-GlcNAcylation-mediated Thr32 in mitosis (Zhang et al., 2011; Fong et al., 2005). Importantly, according to *O*-GlcNAcylation of H3 can competitively reduce the level of H3S10 phosphorylation, but removal of *O*-GlcNAc from H3S10 is required for entering mitosis during the G_2_-M transition phase (Zhang et al., 2011), H3S10 has been considered as a molecular checkpoint for entering mitosis (Van Hooser et al., 1998). Further study confirmed that H3S10 phosphorylation provides a binding platform for the phospho-binding 14-3-3 proteins and histone acetyltransferase MOF to trigger acetylation of histone H4 at lysine 16 (H4K16ac), and H3S10 phosphorylation and H4K16ac further coordinatively regulate the binding site for bromodomain protein BRD4 (Zippo et al., 2009). Moreover, in addition to up-regulation of H3S10 phosphorylation in hepatocellular carcinoma and primary lung cancer (Zhu et al., 2016), there is sufficient evidence to prove that H3S10 phosphorylation is responsible for neoplastic cell transformation and oncogene c-fos/c-Jun activation (Choi et al., 2005), suggesting the important coordinative role between *O*-GlcNAcylation and other histone modifications in tumor development.

### Coordination of *O*-GlcNAcylation and the other chromatin remodelers

In addition to direct glycosylation of histones, OGT can also affect other histones modifications by crosslinking other chromatin remodelers. In line with this view, overexpression of OGT raises global H3K9ac and H3K27me3 level in cells, suggesting that OGT may collaborate with other chromatin remodelers to regulate histone modification (Sakabe and Hart, 2010). Histone H3 is a well-known specific substrate of CARM1 (a co-activator-associated argine methyltrnsferse 1), however the arginine methylation at H3R17 was regulated by *O*-GlcNAc modification of CARM1 (Charoensuksai et al., 2015; Sakabe and Hart, 2010; Schurter et al., 2001). Interestingly, the crosstalk between OGT and chromatin remodelers also exists in DNA damage repair pathway. *O*-GlcNAc-modified H2AX and MDC1 (mediator of DNA damage check point 1) are enriched at DNA damage foci, but suppress the expansion of DNA damage-produced phosphorylation at H2AX Ser139 site on the chromatin (Chen and Yu, 2016). What happens on histone variant H3.3 is another example. OGT regulates histone chaperone HIRA complex via *O*-GlcNAc modification, and subsequently deposits histone variant H3.3 to genic regions, then further governs H3.3 nucleosome assembly and cell senescence (Lee and Zhang, 2016; Ricketts and Marmorstein, 2016). Although the mechanism of targeting of histone H3.3 deposition at specific loci in whole genome remains largely unclear, histone H3.3 has been considered as epigenetic regulator that is dispensable for proper expression of genes (Kato et al., 2015). Furthermore, the function of histone variant H3.3 is also regulated by *O*-GlcNAcylated MLL5 which is a specific histone H3K4 tri-methylase (Gallo et al., 2015). In cells, *O*-GlcNAcylated MLL5 represses histone H3.3 expression and facilitates the self-renewal of adult glioblastoma (Ding et al., 2015; Sebastian et al., 2009). On the other hand, histone H3.3 substitution and mutation are found in several tumors such as bone tumors and glioblastoma (Kato et al., 2015). However, whether substituted and mutated H3.3 is related to OGT-mediated *O*-GlcNAcylation remains to be further investigated.

### Crosstalk of *O*-GlcNAcylation and epigenetics in cancer

Intracellular unbalanced epigenetic changes or abnormal *O*-GlcNAcylation levels can lead to various diseases. However, the evidence of crosslink between epigenetic changes and *O*-GlcNAcylation in tumorigenesis is gradually increased. For example, in addition to modulating the activity of deacetylase SIRT1 in an AMPK-dependent manner in breast cancer cells (Ferrer et al., 2017), OGT-mediated *O*-GlcNAcylation can also affect cancer cell growth and invasion through regulating the oncogenic transcription factor FOXM1 (Caldwell et al., 2010), suggesting the interplay of *O*-GlcNAcylation and SIRT1 in cancer cells. Another deacetylase HDAC1 also can be *O*-GlcNAc modified to promote its phosphorylation level, and simultaneously enhances its enzyme activity. This *O*-GlcNAc modified HDAC1 further functions in regulating the global histone acetylation level, and therefore influences the cell proliferation, invasion, and migration by regulating target genes in HepG2 cells (Zhu et al., 2016), suggesting the potential coordinative role in hepatic carcinoma. Given that abnormal histone acetylation has been implicated in tumorigenesis, the crosslink mechanism of *O*-GlcNAcylation and histone acetylation might provide a new direction for cancer therapy. On the other hand, OGT can modulate the integrity of multi-protein complex through *O*-GlcNAcylation-mediated subunit protein stability. For instance, OGT-mediated *O*-GlcNAcylation of Ezh2 at Ser75 stabilizes the PCR2 complex, which further promotes histone H3K27 tri-methylation in breast cancer MCF-7 cells (Chu et al., 2014). Taken together, the collaborative effects of OGT with histones and other non-histone proteins have been involved in gene transcription and tumorigenesis. Therefore, figuring out the mechanism of the network between OGT and epigenetic changes in tumorigenesis will provide a novel theoretical basis for cancer research.

## Conclusion and perspectives

**Figure 4:**
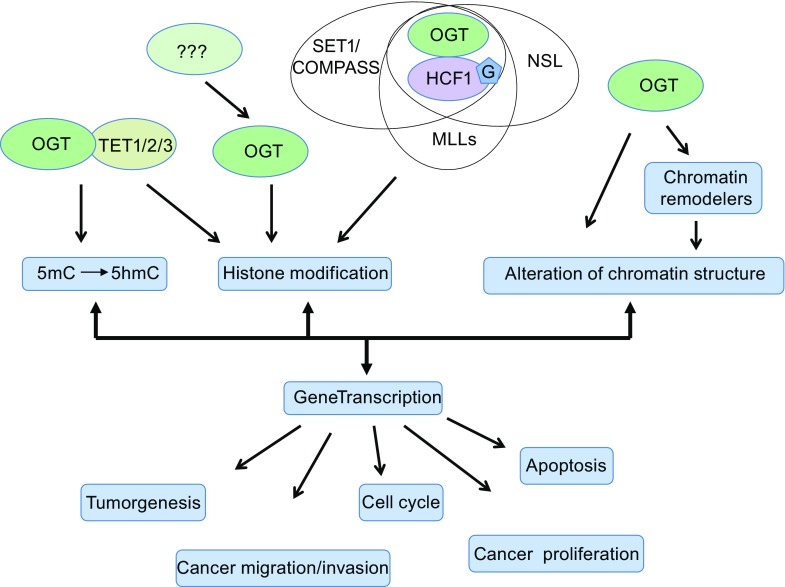
**Schematic diagram of coordination between OGT-mediated** ***O*** **-GlcNAcylation and chromatin remodelers in intracellular fundamental functions**

In summary, as the only intracellular mono-*O*-GlcNAc transferase, OGT has been implicated in fundamental intracellular biological functions through regulating cellular metabolism, chromatin structure, and gene transcription by cooperating with epigenetic mechanism. Although the precise regulation mechanism of network between OGT-mediated *O*-GlcNAcylation and epigenetic changes still remains largely unclear and to be further investigated, increasing evidence has proven that OGT-mediated *O*-GlcNAcylation and epigenetic changes form a complex network to further control the gene expression and signaling pathways. This coordination between *O*-GlcNAcylation and epigenetic changes contributes a critical role in initiation and progression of many human diseases (Fig. 4).
